# Supplementary material for: The Prognostic Value of Pulmonary Hypertension in Patients with Mitral Regurgitation Undergoing Mitral Valve Transcatheter Edge-to-Edge Repair: A Systematic Review and Meta-Analysis
Source: Diagnostics (Basel). 2025 Mar 27;15(7):852. doi: 10.3390/diagnostics15070852 (PMC11988427; doi:10.3390/diagnostics15070852)
Supplement: Supplementary file 1 [file diagnostics-15-00852-s001.zip › diagnostics-3355955-supplementary.pdf]

Supplementary material

Table S1. Risk of bias assessment of the included studies according to Newcastle-Ottawa Scale (NOS).

First Author	Selection overall				Comparability	Outcome overall			Overall score
	Representativeness	Selection of non-exposed	Exposure ascertainment	Outcome absent at baseline		Outcome assessment	Length of follow-up	Adequacy of follow-up	
Ubben, T. 2024	1	1	1	1	1	1	NA	1	7
Yoon, S. H. 2022	1	1	NA	1	1	1	1	1	7
Iliadis, C. 2022	1	1	NA	1	NA	1	1	1	6
Tanaka, S. 2021	1	1	1	1	NA	1	1	NA	6
Ben-Yehuda, O. 2020	1	1	1	1	1	1	1	1	8
Al-Bawardy, R. 2020	1	1	1	1	1	1	1	1	8
Rashi, Y. 2020	1	1	1	1	1	1	1	1	8
Geyer, M. 2020	1	1	1	1	1	1	1	1	8

Debonnaire, P. 2020	1	1	1	1	NA	1	1	1	7
Kreusser, M. 2019	1	1	1	1	NA	1	1	1	7
Adamo, M. 2019	1	1	NA	1	1	1	1	1	7
Buzzatti, N. 2019	1	1	1	1	NA	1	1	1	7
Tigges, E. 2018	1	1	1	1	1	1	1	1	7
Kalbacher, D. 2017	1	1	1	1	1	1	1	1	8
Buccheri, S. 2017	1	1	1	1	NA	1	1	1	7
Saji, M. 2017	1	1	1	1	NA	1	1	1	7
Yzeiraj, E. 2017	1	1	1	1	1	1	1	1	7
Puls, M 2014	1	NA	1	1	1	1	1	1	7
Matsumoto, T. 2014	1	1	1	1	NA	1	1	1	8

Figure S1. Research strategy.

PubMed: 271 **Cochrane Library: 35.** **Embase: 635**

Search	Actions	Details	Query	Results	Time
#5			Search: (((Pulmonary hypertension[MeSH Terms]) OR (hypertension, pulmonary[MeSH Terms])) OR ((((((Arterial Hypertension, Pulmonary[Title/Abstract]) OR (Hypertension, Pulmonary Arterial[Title/Abstract])) OR (pulmonary artery hypertension[Title/Abstract])) OR (pulmonary hypertension[Title/Abstract]))) OR ((PH[Title/Abstract]) OR (PHT[Title/Abstract]))))) AND ((((((((((Transcatheter mitral valve repair[Title/Abstract]) OR (percutaneous edge-to-edge repair[Title/Abstract])) OR (Transcatheter repair[Title/Abstract])) OR (percutaneous mitral valve repair[Title/Abstract])) OR (transcatheter edge-to-edge repair[Title/Abstract])) OR (MitralClip[Title/Abstract])) OR (mitral valve repair[Title/Abstract])) OR (transcatheter repair[Title/Abstract])) OR (percutaneous repair[Title/Abstract])) OR ((TMVR[Title/Abstract]) OR (TEER[Title/Abstract])))	271	20:01:34
#4			Search: (((((((((Transcatheter mitral valve repair[Title/Abstract]) OR (percutaneous edge-to-edge repair[Title/Abstract])) OR (Transcatheter repair[Title/Abstract])) OR (percutaneous mitral valve repair[Title/Abstract])) OR (transcatheter edge-to-edge repair[Title/Abstract])) OR (MitralClip[Title/Abstract])) OR (mitral valve repair[Title/Abstract])) OR (transcatheter repair[Title/Abstract])) OR (percutaneous repair[Title/Abstract])) OR ((TMVR[Title/Abstract]) OR (TEER[Title/Abstract]))	10,464	20:01:18
#3			Search: ((Pulmonary hypertension[MeSH Terms]) OR (hypertension, pulmonary[MeSH Terms])) OR ((((((Arterial Hypertension, Pulmonary[Title/Abstract]) OR (Hypertension, Pulmonary Arterial[Title/Abstract])) OR (pulmonary artery hypertension[Title/Abstract])) OR (pulmonary hypertension[Title/Abstract]))) OR ((PH[Title/Abstract]) OR (PHT[Title/Abstract]))))	606,193	20:00:46
#2			Search: (((((Arterial Hypertension, Pulmonary[Title/Abstract]) OR (Hypertension, Pulmonary Arterial[Title/Abstract])) OR (pulmonary artery hypertension[Title/Abstract])) OR (pulmonary hypertension[Title/Abstract]))) OR ((PH[Title/Abstract]) OR (PHT[Title/Abstract]))	591,325	20:00:08
#1			Search: (Pulmonary hypertension[MeSH Terms]) OR (hypertension, pulmonary[MeSH Terms])	42,993	19:59:29

Cochrane Library

Advanced Search

Search manager

Save this search | View/Share saved searches | ? Search help

Print search history

#	Query				Results
#1	Pulmonary Arterial Hypertension	S	MeSH	Limits	3118
#2	(pulmonary hypertension):ab,ti,kw OR (pulmonary arterial hypertension):ab,ti,kw OR (pulmonary artery hypertension):ab,ti,kw			Limits	6225
#3	#1 OR #2			Limits	6517
#4	(transcatheter mitral valve repair):ab,ti,kw OR (percutaneous mitral valve repair):ab,ti,kw OR (transcatheter edge-to-edge repair):ab,ti,kw OR (MitraClip):ab,ti,kw OR (percutaneous edge-to-edge repair):ab,ti,kw OR (TEER):ab,ti,kw OR (TMVR):ab,ti,kw OR (transcatheter repair):ab,ti,kw OR (percutaneous repair):ab,ti,kw OR (mitral valve repair):ab,ti,kw			Limits	1046
#5	#3 and #4			Limits	35

Clear all

Highlight orphan lines

Embase

Embase session results (6 Mar 2024)

No.	Query	Results
#5	#3 AND #4	635
#4	'transcatheter mitral valve repair'/exp OR 'transcatheter mitral valve repair':ab,ti OR 'percutaneous mitral valve repair':ab,ti OR 'transcatheter edge-to-edge':ab,ti OR 'mitraclip':ab,ti OR 'percutaneous edge-to-edge':ab,ti OR 'teer':ab,ti OR 'tmvr':ab,ti OR 'transcatheter repair':ab,ti OR 'percutaneous edge-to-edge repair':ab,ti OR 'transcatheter edge-to-edge repair':ab,ti OR 'percutaneous repair':ab,ti OR 'mitral valve repair':ti,ab	17022
#3	#1 OR #2	134255
#2	'pulmonary arterial hypertension':ab,ti OR 'pulmonary artery hypertension' OR 'pulmonary hypertension':ab,ti	92941
#1	'pulmonary hypertension'/exp	124139

Copyright © 2024 Elsevier Limited except certain content provided by third parties.

Embase is a trade mark of Elsevier Life Sciences IP Limited.

RELX™

file:///Users/shancuoji/Desktop/queries.html

Figure S2. Funnel plot by publication bias test.

A.

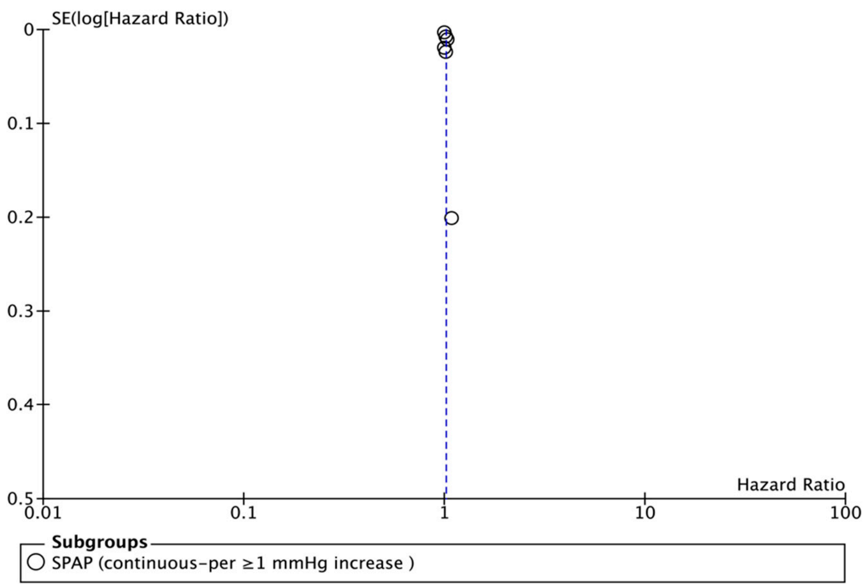

B.

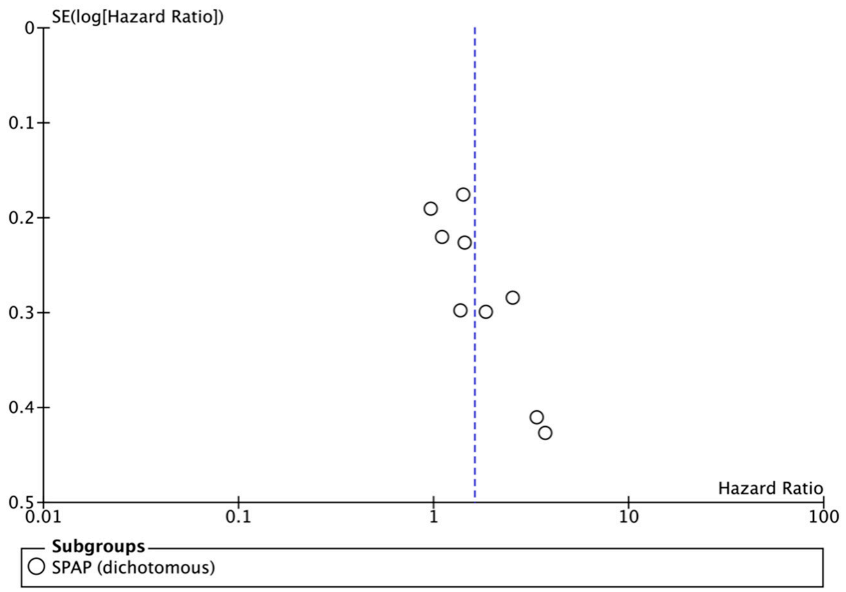

Figure S3. Pooled standard MD of SPAP before and after M-TEER within 6-month follow-up.

Study or Subgroup	after M-TEER Mean	after M-TEER SD	after M-TEER Total	before M-TEER Mean	before M-TEER SD	before M-TEER Total	Weight	Std. Mean Difference IV, Random, 95% CI
**SPAP (Subgroup=mild or moderate PH)**								
Rashi, Y. 2020	48.4	14.4	96	48.9	6.2	96	18.6%	−0.04 [−0.33, 0.24]
Tigges, E. 2018	40.5	9	227	44	6	227	19.8%	−0.46 [−0.64, −0.27]
**Subtotal (95% CI)**			**323**			**323**	**38.4%**	**-0.27 [-0.67, 0.14]**
Heterogeneity: $Tau^2 = 0.07$; $Chi^2 = 5.68$, df = 1 ($P = 0.02$); $I^2 = 82\%$								
Test for overall effect: Z = 1.29 ($P = 0.20$)								
**SPAP (Subgroup=severe PH)**								
Rashi, Y. 2020	57.5	16	59	70.9	9.3	59	16.9%	−1.02 [−1.40, −0.63]
Tigges, E. 2018	49.5	13.4	216	61	8.2	216	19.7%	−1.03 [−1.23, −0.83]
**Subtotal (95% CI)**			**275**			**275**	**36.6%**	**-1.03 [-1.21, -0.85]**
Heterogeneity: $Tau^2 = 0.00$; $Chi^2 = 0.01$, df = 1 ($P = 0.94$); $I^2 = 0\%$								
Test for overall effect: Z = 11.34 ($P < 0.00001$)								
**SPAP (Subgroup=IPC-PH)**								
Mandurino-Mirizzi, A. 2021	48.2	15.8	13	53.6	15.5	13	10.7%	−0.33 [−1.11, 0.44]
**Subtotal (95% CI)**			**13**			**13**	**10.7%**	**-0.33 [-1.11, 0.44]**
Heterogeneity: Not applicable								
Test for overall effect: Z = 0.84 ($P = 0.40$)								
**SPAP (Subgroup=CPC-PH)**								
Mandurino-Mirizzi, A. 2021	55.1	15.8	27	60.6	15.6	27	14.3%	−0.35 [−0.88, 0.19]
**Subtotal (95% CI)**			**27**			**27**	**14.3%**	**-0.35 [-0.88, 0.19]**
Heterogeneity: Not applicable								
Test for overall effect: Z = 1.26 ($P = 0.21$)								

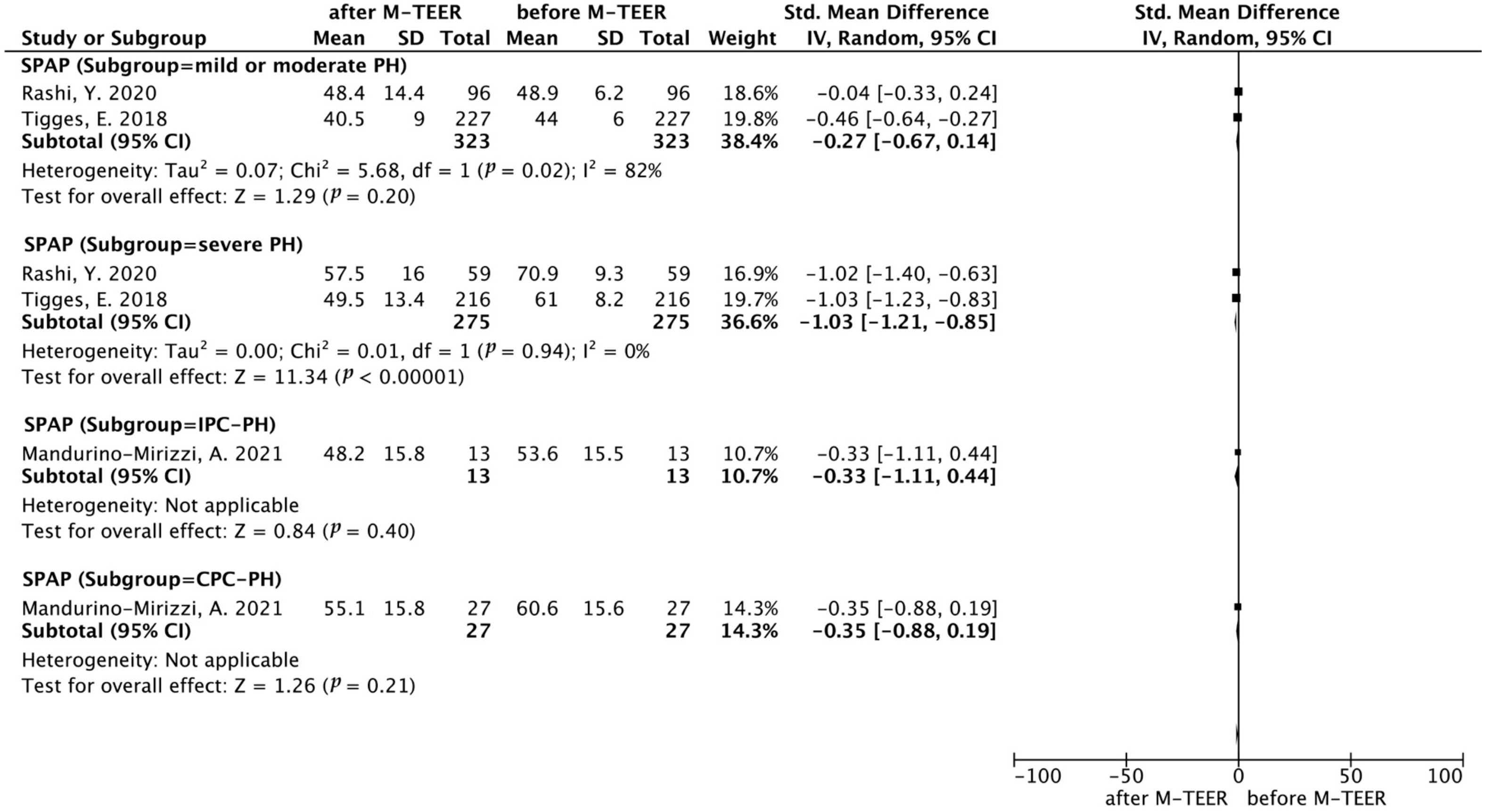

Abbreviations: CI- Confidence interval; IV- inverse variance; MD-Mean difference
